# Plausibility of stromal initiation of epithelial cancers without a mutation in the epithelium: a computer simulation of morphostats

**DOI:** 10.1186/1471-2407-9-89

**Published:** 2009-03-23

**Authors:** Stuart G Baker, Ana M Soto, Carlos Sonnenschein, Antonio Cappuccio, John D Potter, Barnett S Kramer

**Affiliations:** 1 Biometry Research Group, Division of Cancer Prevention, National Cancer Institute, Bethesda, USA; 2 Department of Anatomy and Cell Biology, Tufts University School of Medicine, Boston, USA; 3 Bioinformatics and Computational Systems Biology of Cancer, Institut Curie, Paris, France; 4 Division of Public Health Sciences, Fred Hutchinson Cancer Research, Seattle, USA; 5 Office of Disease Prevention, National Institutes of Health, Bethesda, USA

## Abstract

**Background:**

There is experimental evidence from animal models favoring the notion that the disruption of interactions between stroma and epithelium plays an important role in the initiation of carcinogenesis. These disrupted interactions are hypothesized to be mediated by molecules, termed morphostats, which diffuse through the tissue to determine cell phenotype and maintain tissue architecture.

**Methods:**

We developed a computer simulation based on simple properties of cell renewal and morphostats.

**Results:**

Under the computer simulation, the disruption of the morphostat gradient in the stroma generated epithelial precursors of cancer without any mutation in the epithelium.

**Conclusion:**

The model is consistent with the possibility that the accumulation of genetic and epigenetic changes found in tumors could arise after the formation of a founder population of aberrant cells, defined as cells that are created by low or insufficient morphostat levels and that no longer respond to morphostat concentrations. Because the model is biologically plausible, we hope that these results will stimulate further experiments.

## Background

Cell-to-cell and/or tissue-to-tissue communication is crucial for tissue organization. Its disruption can play an important role in the initiation of cancer [1,2]. These communications involve substances analogous to a morphogen in the developing embryo that diffuse through the tissue creating a concentration gradient. Local concentrations of these substances influence the phenotype of neighboring cells. These substances have been called morphostats [3,4]. Various models have been proposed to explain how the concentration gradient of morphogens generates differentiated tissues during embryogenesis [3]; these models could be applied to examine the role of morphostats in carcinogenesis but, for our purpose here, we assume a simple model in which the source of the morphostat gradient lies in the stroma.

One suggestive piece of evidence for the existence of morphostats is the development of tumors when foreign bodies, which serve as barriers to potential diffusion, have been inserted subcutaneously in mice. Early experiments found a carcinogenic response for various chemically inert substances inserted subcutaneously, but only when they were implanted intact and not in powdered form [5]. If there were chemically induced genetic changes, the rate of tumor formation from a foreign-body implant in powdered form would be the same or greater than the rate of tumor formation from a solid implant of the same mass (and less surface area). Because this was not the case, it is highly doubtful that there was a chemically-induced genetic initiation (like a mutation). Without genetic initiation, it is unlikely that an intact implant would have the role of a promoter. Later experiments involving the subcutaneous insertion of a Millipore filter showed that tumors formed only when the pores in the filter were sufficiently small [6], suggesting a threshold size of pore that permits morphostat diffusion. An alternative explanation for the initiation of tumors in foreign-body experiments is that inflammatory responses lead to mutations that cause cancer. However, this explanation does not fit the fact that inflammation (specifically, filter invasion by cytoplasmic process and phagocytic and lysosomal activity) was associated with large pore implants when almost no tumors were observed, but was not associated with small pore implants when many tumors were observed [6]. Thus, because an explanation for this peculiar phenomenon based on an initiating event involving genetic mutations appears unlikely, we have recently identified "foreign-body" carcinogenesis as a key paradox in the initial steps of carcinogenesis if one were to adopt the explanation suggested by the somatic mutation theory [7].

Other evidence consistent with the role of morphostats in carcinogenesis stems from transplantation experiments in which tumors arise when normal epithelial cells were transplanted next to stromal cells in rats treated with either a physical carcinogen [8] or a chemical carcinogen with a very short half-life to reduce the chance of indirect exposure of the epithelial cells to the carcinogen [9].

Even though some putative morphostats have been identified [3], the full spectrum is, as yet, unknown, as are the mechanisms by which they influence carcinogenesis. Based on the prevalent somatic mutation theory, one hypothesis would be that at least two mutations in genes regulating cell proliferation are required: one in an epithelial cell and one in a surrounding stromal cell [10]. Recent data showing lack of evidence for mutations in the stroma of tumors argues against this option [11]. An alternative hypothesis is that the perturbation in a morphostat gradient could initiate carcinogenesis without any requirement for a mutation. To investigate this latter hypothesis, we developed a simple mathematical model (computer simulation) of the process of cell renewal, the diffusion of a morphostat, and the effect of morphostat concentrations on cell phenotype. We then mathematically perturbed the model to simulate disruption of the morphostat gradient arising from a block in the stroma.

Our goal was to determine whether or not disruption of a morphostat gradient is sufficient to create aberrant cells in the context of cell renewal without the need to postulate a mutation in the epithelial cells to initiate the development of cancer. We define an aberrant cell as an epithelial cell created by a low morphostat level, the phenotype of which is no longer under the control of that morphostat gradient. Because these aberrant cells are unable to respond to a morphostat, we view them as precursors of cancer that have the potential to obtain a selective advantage as a result of higher proliferation rates or a propensity toward genetic or epigenetic aberration, or some combination of these.

If our mathematical model provides evidence that aberrant cells in a dynamic setting of cell renewal can indeed be generated without mutations, it would provide impetus for new research directions in carcinogenesis and, specifically, attempts at empiric experimental testing under a paradigm that is quite different from the dominant somatic mutation theory.

Most mathematical models of carcinogenesis [12-14] begin with a nascent tumor cell. In contrast our model begins with normal tissue. Our model is related to mathematical models for morphogenesis in that it depends on diffusion. The classic mathematical model for morphogenesis was a set of differential equations proposed by AM Turing [15] in a 1952 paper in which he coined the word "morphogen." Turing, who was a computer pioneer and a cryptologist, noted, then, the future possibility of modeling morphogen diffusion using a digital computer. A cell polarity model of carcinogenesis [14] also involves morphogens, but the morphogens in that model play a very different role from those in our model, in that they induce a loss of cell polarity leading to division in an abnormal direction rather than influencing tissue phenotype.

## Methods

Our model focuses on the effect of morphogens on tissue architecture. The two-dimensional mathematical model of the tissue consists of two arrays of numbers, each corresponding to a grid of rectangles, one for cell type and one for morphostat levels.

### Cell type

The first array of numbers codes for cell type, displayed as different colored rectangles. Some rectangles represent biological cells, sometimes called *in silico *cells [12,13]. Other rectangles represent the lumen as would be found in hollow organs or the free surface as in the skin. Lastly, some rectangles represent what we call open spaces, which are mathematical indications of room for cells to move during the tissue-renewal process. The word "cell" has a mathematical meaning as a quantity governed by rules involving geometrically adjacent quantities The mathematical term "cellular automata" refers to an array of numbers whose "life processes" are governed by simple mathematical rules related to nearby cells [16].

**Figure 1:**
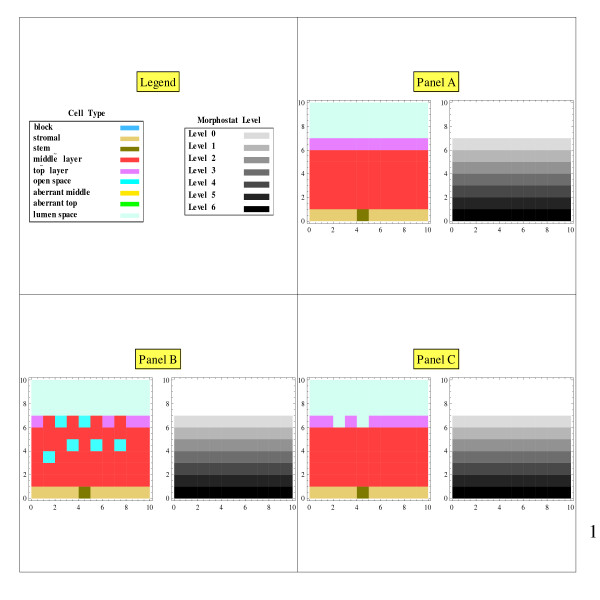
**No block in the stroma: snapshots**. Panel A shows stable tissue architecture and a stable morphostat gradient. In Panel B some top-layer cells have sloughed off and were replaced by middle layer cells, and some middle layers cells have moved upward leaving open spaces. In Panel C, the middle layer cells on top have become top layer cells because they experienced the threshold Level 0 morphostat concentration; also open spaces on top have been classified as lumen.

**Figure 2:**
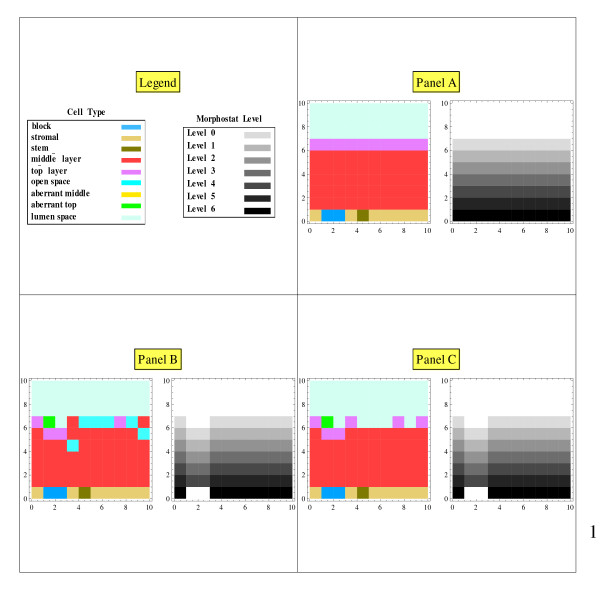
**Solid block in the stroma: snapshots**. Panel A shows stable tissue architecture and a stable morphostat gradient when a block has been inserted in the stroma. In Panel B some top-layer cells have sloughed off and were replaced by middle layer cells, some middle layers cells have moved upward leaving open spaces, and one top layer cell has become aberrant due to experiencing a morphostat level below the Level 0 threshold. In Panel C, middle layer cells on top have become top layer cells due to reaching the threshold morphostat Level 0.

The initial grid of cell type**s **displays a structured epithelial tissue above a layer of stromal cells (Figure 1, Panel A, left). The epithelial tissue consists of (a) a stem cell centered just below the bottom layer, (b) middle-layer cells on either side of the stem cell and in layers above the stem cell, and (c) a top-layer of cells below the lumen space. The term "stem cells" here refers to the normal stem cells that generate tissue renewal, and not to cancer stem cells. Regarding the controversy about whether stem cells are defined by their "niche" (i.e., their microenvironment) or its alternative, namely, that "stemness" is an intrinsic cellular property, we subscribe to the former. However, in the context of this simulation, these hypotheses are equivalent. A block can be mathematically introduced in the stroma layer (Figure 2, Panel A, left).

### Morphostat level

The second array of numbers codes for morphostat level displayed as shaded rectangles on a gray scale. The initial morphostat gradient ranges from the highest concentration at the bottom layer of the epithelium (just above the stromal cells) and falls to the threshold level at the top layer (Figure 1, Panel A, right). The threshold level defines the morphostat concentration in middle-layer cells at which differentiation occurs. The initial threshold level of the morphostat concentration is based on the initial tissue architecture. The highest morphostat concentration is at the bottom layer and set to level 6. Therefore, the initial morphostat concentration at the top layer, which is six cells away, is set to 0. Level 0 is thus set as the threshold level for a middle-layer cell to become a top-layer cell.

### Cell renewal model

We model biological cell renewal using various rules for the movement and differentiation (change of phenotype) of cells based on the type of cell, the type of adjacent cells, and the strength of the morphostat gradient at an adjacent cell. (An adjacent cell is defined as a cell above, below, or lateral to any particular cell). There is only one parameter specifying a probability distribution function, namely the probability of sloughing of a top layer cell. The remaining changes are simple functions of cell type and morphostat level.

The first three rules are motivated by findings summarized in LeBlond [17] that "the cells of the intestinal epithelium migrate from the crypts along the villus surface to the villus tips where they fall to their death in the lumen, that the alveolar cells of the lung migrate out the alveolar tissues to the alveolar spaces and from the bronchia and trachea, to fall into the esophagus, and finally that lymphocytes leave the thymus in large numbers to enter the blood circulation." This cell renewal process has been postulated to arise from cell pressure arising from cell division in the basal layer [17]. Using the following three rules, we model this pressure as open space moving from the top layer to the stem cell, which translates into cell movement from the stem cell to the top layer.

#### Rule 1. Sloughing off

If a top-layer cell is below a lumen space, it sloughs off with a given probability and creates an open space in its former position.

#### Rule 2. Upward cell movement

If a middle-layer cell is below an open space, it moves upward into the open space, creating an open space in its former position.

#### Rule 3. Lateral cell movement

If a middle-layer cell is lateral to an open space, if upward cell movement into the open space is not possible, and if there is no stem cell below the open space, a middle-layer cell adjacent to the open space moves laterally into the open space (and toward an imaginary vertical line at the horizontal position of the stem cell), creating an open space in its former position.

Rule 1 is based on the statement in LeBlond [17] that "an important feature of renewal systems is the existence of an outlet allowing eventual elimination of cells produced. Thus, the differentiated cells arising in epithelia are cast off to the outside or into a lumen, the blood cells formed in hematopoietic organs pass into circulation, and so on." Rules 2 and 3 mimic cell migration in the renewing tissue. In our model, the major movement is upward with an additional lateral movement from the stem cell when upward movement is not possible. Cell renewal also involves stem cell division summarized as follows.

#### Rule 4. Stem-cell division

If a stem cell is adjacent to an open space, the stem cell undergoes division to generate both a middle-layer cell to fill the open space and a new stem cell at the original fixed location.

Rule 4 is based on three statements in LeBlond [17]: (i) "in most renewal systems...division is chiefly seen in cells that are little or not differentiated, the stem cells," (ii) "mitoses of stem cells have to provide a continuous supply of differentiated cells, while maintaining their own stock," and (iii) "when single cells are considered, the steady state implies that, for any cell lost from the population, another cell must divide and thus make up the loss. The simplest way for nature to achieve this aim would be for each division of a stem cell to produce one daughter cell that differentiates and another one that remains the stem cell."

Another rule is needed for changes in the morphostat concentration.

#### Rule 5. Morphostat changes

The morphostat concentration in a mathematically defined cell (which would be on the outside of a physical cell) equals the maximum of the morphostat concentrations in the adjacent cells (excluding open space) minus one.

Rule 5 is based on an experimentally supported theory that morphogen diffusion occurs through the extracellular matrix [18,19]. The effect of morphostats on cell phenotype is modeled by Rules 6 and 7.

#### Rule 6. Differentiation

A middle-layer cell becomes a top-layer cell if the morphostat concentration drops to a threshold level.

Rule 6 is based on (i) a study of the rat esophagus where numerous radioactively labeled spinous layer cells appeared two days after basal cells were radioactively labeled, suggesting a phenotypic change after cell movement to the spinous layer [20] and (ii) the experimental result that a cell can "read its position in concentration gradient without reference to its neighbors" [21]. The premise that the morphostat concentration is causing a phenotypic change is more speculative

#### Rule 7. Generation of aberrant cells

If the morphostat concentration of a middle-layer (top-layer) cell drops below the threshold level, the cells become an aberrant middle-layer (top-layer).

Rule 7 is the key speculation. The purpose of the model is to determine how Rule 7 fares in the context of cell renewal.

These rules have been summarized in a precise mathematical formulation [see Additional file 1] and implemented in software written in Mathematica, Version 7 [22] [see Additional file 2].

### Application of Rules

We successively applied Rules 1 to 4 from the top to the bottom rows of cells. Within each row, we applied the rules from the middle to the outside, but we also investigated applying them from the outside to the middle. For the layer above the stem cell, we repeated the rules a number of times equal to half the width of the plot in order to complete the cell-renewal cycle within a round. We used a sloughing probability of 0.3 but also investigated other sloughing probabilities.

After the aforementioned round of cell movements, we renamed any open space below the lumen space as a lumen space. Then we evaluated the morphostat concentration at each cell according to Rule 5. The process mimics passive diffusion from higher to lower morphostat concentrations and determines cell-fate change across the morphostat gradient. We applied Rule 5 from top to bottom and left to right in the grid (also considering right to left), repeating this procedure once to ensure sufficient update. Then we mathematically changed the cell phenotypes according to Rules 6 and 7.

**Figure 3:**
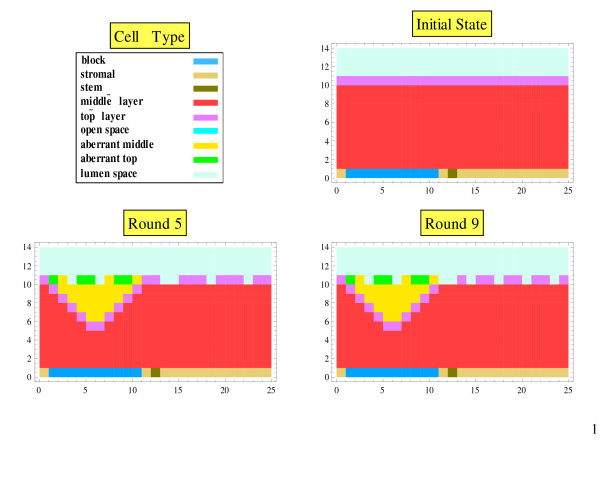
**Solid block in the stroma: multiple rounds**. The Initial State represents stable tissue architecture when a solid block is first inserted into the stroma. A round is one cycle of cell movement and morphostat update through all the cells. At Round 5 a mass of aberrant cells forms above top layer cells, which persists through later rounds.

**Figure 4:**
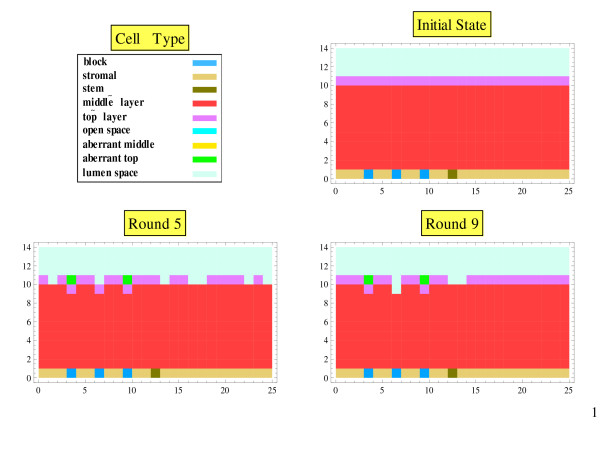
**Perforated block in the stroma: multiple rounds**. The Initial State represents stable tissue architecture when a solid block is first inserted into the stroma. A round is one cycle of cell movement and morphostat update through all the cells. At Round 5 scattered aberrant cells form above top layer cells, which persists through later rounds.

The disruption of the morphostat gradient was created by assigning an unchanging negative value for the morphostat concentration in some stroma cells bordering the epithelial cells. When the morphostat concentrations were updated, these negative values mathematically prevented the morphostat concentration in the stroma from directly influencing the morphostat concentration of the epithelial cells on the other side of the block, which is the mathematical equivalent of preventing diffusion across the block. We considered both a solid block (Figure 3) and a perforated block (Figure 4). The analogy to the foreign body experiments involving implants is clear. As a check of stability, temporary initial perturbations in the morphostat gradient were also investigated.

## Results

With no disruption of the morphostat gradient, the tissue was in equilibrium under cell renewal (Figure 1): the cell layers and the morphostat gradient remained unchanged from the initial conditions of the model. With the mathematical block in the stroma, the morphostat concentration in the stroma indirectly influenced the morphostat concentration in the epithelial cells on the other side of the blockage through a chain of cells around the blockage, at which point the morphostat concentration was reduced. Due to the lower morphostat concentrations when the morphostat gradient was blocked, aberrant cells were generated (Figure 2). With a solid morphostat block, a sizeable number of aberrant cells were created (Figure 3). With a perforated block, only a few aberrant cells were created (Figure 4).

The results were not qualitatively dependent on (i) whether cell renewal was evaluated from middle to outside or vice versa; (ii) the number of repeated evaluations of cell renewal in the layer above the stem cells (although with only a few repeats open space remained at the end of a round); (iii) the sloughing probability; and (iv) whether the morphostat gradient was evaluated from left to right or right to left. Initial temporary perturbations in the morphostat field disappeared although sometimes they resulted in the creation of aberrant cells. The main observation from varying aspects of the simulation (which was not obvious beforehand) was that the cell renewal process had little impact on the development of aberrant cells, which was primarily driven by the morphostat gradient.

## Discussion

In the model of carcinogenesis described here, the aberrant cells remain in the tissue and may still exercise their constitutive ability to proliferate leading to the histoarchitectural disturbances present in cancers [1-3]. The lack of a DNA mutation in the epithelial cells as a necessary initial event in carcinogenesis challenges the tenets of the somatic mutation-theory of carcinogenesis [23]. However, our model does not preclude that mutations could subsequently arise due to cytoarchitectural dislocations in the aberrant cells. In support of this view, Prehn [24] argued that cancer might cause mutations rather than vice versa. Consistent with this line of thought, the aberrant cells could become a founder population for cells that may undergo genetic and epigenetic changes: mutations may still play a role after the initiation of cancer by disruption of morphostats. Because, in our simulations, the perturbation of the morphostat gradient by the solid block creates more aberrant cells than by the perforated block, the chances for subsequent genetic and epigenetic changes may be greater with the former.

Perhaps the strongest experimental evidence for the existence of aberrant cells created by low morphostat levels are foreign-body implant experiments investigating preneoplastic cells (that could be either aberrant cells or cells derived from aberrant cells). By transferring implants from one rat to another, Brand et al. showed that preneoplastic cells first appear on the implant film and then in the capsule surrounding the film [25]. Using radioactive bone marrow, Barnes et al. showed that the preneoplastic cells in foreign-body experiments could not have arisen in the bone marrow [26], partly leading to the belief that the preneoplastic cells could have originated from pericytes around local capillaries [27]. More experimental work is needed, perhaps involving the use of microarray assessment of foreign-body experiments [7].

Our simulation provides evidence that disruption of morphostats in the stroma could create aberrant epithelial cells without the need to invoke an initiating mutation in those epithelial cells. Rule 7, which states that low morphostat levels induce aberrant cells, is certainly critical for the model. However we did not know in advance whether or not Rule 7 would lead to aberrant cells in the context of cell renewal. Also, the results for the perforated versus the solid block were not obvious.

### Biological nature of morphostats

We did not need to specify the biological nature of a morphostat, though we draw a parallel with established morphogen gradients in embryogenesis. Potter predicted that Wnt/wingless protein could be a morphostat [10] and subsequently noted that there is considerable support for this view [3,28-31].

### Morphostat disruption and known carcinogens

It is important to emphasize that our model does not require specifying the cause of the disruption of the morphostat gradient. Possible causes could include physical (radiation), biological (infectious agents), chemical carcinogens and/or foreign bodies inserted in the stroma.

Although radiation is known to cause DNA damage, various studies suggest that radiation may initiate carcinogenesis via disruption of cell communication [8,31]. Radiation can induce genetic changes in neighboring cells that receive no direct radiation (the "bystander effect") but are affected through cell-to-cell communication [32]. Bone marrow cells irradiated with α-particles *in vitro *and transplanted into mice induced chromosomal instability up to one year after transplantation [33].

A major risk factor for hepatocellular carcinoma is chronic infection by hepatitis B and C viruses. Although the cellular mechanisms of hepatocarcinogenesis are poorly understood, recent work has pointed to dysregulation of the WNT/Frizzled receptor elements (which, as mentioned previously, might be related to morphostats) as one of the most common and earliest event in hepatocarcinogenesis [34].

Mouse salivary glands infected with polyoma virus (PV) had different incidences of cancer depending on the stage of development, suggesting that the neoplastic response to PV is related to morphogenesis. Moreover when epithelial and mesenchymal components were separated, transplanted, and then infected by PV, neither component separately gave rise to tumors. Instead, tumors did arise when the epithelial and mesenchymal components were combined prior to transplantation and infection by PV [35].

Some proven human carcinogens are not known to be genotoxic. For example, tamoxifen, a selective estrogen receptor modifier, affects growth factor action but is not genotoxic [36]; similarly, the human carcinogen ethanol has no known genotoxic capacity [37].

### Critique of the evidence supporting the somatic-mutation theory

For a balanced presentation, it is important to discuss the evidence in favor of the somatic-mutation theory. We present seven arguments used to support the somatic mutation theory and offer some alternative explanations related to the theory that the initiating event is a disruption of the morphostat gradient which then may be followed by genetic instability. The first four arguments rely on a recent review article on oncogenes and cancer [38] from which we quote extensively.

(1) "The first evidence that cancer arises from somatic genetic alterations came from studies of Burkitt's lymphoma, in which one of three different translocations juxtaposes an oncogene, MYC.... Since every malignant lymphocyte carries the MYC translocation, deregulation of the MYC oncogene is probably the initiating event [38]." An alternative interpretation is that the MYC translocation indicates a late-stage event in carcinogenesis after the disruption of the morphostat gradient.

(2) "Second, transfection experiments have shown that mouse fibroblasts, when transfected in vitro with DNA from human cancer cells, acquire some of the properties of malignant cells (i.e. transformation). The transforming activity of the DNA was traced to a human homologue of the retroviral RAS oncogene [38]." An alternative explanation is that the transforming activity *in vitro *would be manifested *in vivo *as a disruption in morphostat gradients.

(3) "Third, the cloning and characterization of the chromosomal breakpoints that are characteristic of follicular lymphomas, and some diffuse large B-cell lymphomas have shown a juxtaposition of the BCL2 oncogene to enhancer elements in the immunoglobulin heave-chain locus, resulting in deregulation of BCL2 [38]." An alternative explanation is that these chromosomal breakpoints occur after cancer initiation caused by disruption of morphostats and their frequency increases due to selection after the carcinogenesis process is underway.

(4) "Fourth, in transgenic mice that carry an activated oncogene from a human tumor, cancers develop that resemble the human tumor. That these cancers appear only after a latent period suggests that alterations in other genes must occur before progression to frank neoplasia and occur – activation of a particular oncogene seems to be necessary but not sufficient for the development of cancer [38]." An alternative explanation is that a single or multiple oncogene(s) disrupt(s) the morphostat gradient, which is consistent with the latent period and the additional observation [39] in this study of transgenic mice that tumors were not observed in every organ in which the oncogene was aberrantly expressed.

(5) An additional argument in support of the somatic mutation theory comes from animal experiments involving the formation of tumors after the insertion of oncogenes into epithelial cells. In one instance, Bradbury et al. [40] introduced the neu/erB-2 oncogene into mouse epithelial cells by infecting cultures of mammary epithelium with the retroviruses containing the oncogene, and then injected 30 to 100 thousands cells into mammary fat pads of mice from which the epithelium had been removed. If no oncogene was inserted, normal epithelium developed, but with the oncogene inserted a variety of abnormal epithelial patterns arose. Remarkably, the tumor incidence was rather low under this protocol (only one adenocarcinoma in 43 injected mice), whereas a variety of infrequent epithelial lesions were recorded in those mammary gland transplants, including only four carcinomas in situ in 43 animal hosts. (See their Table 1). Equally noteworthy, the prominent feature in the whole mounts reproduced in the manuscript was the presence of abundant, apparently normal, ductal mammary tissue in the cleared fat pads injected with neu/erB2 oncogene-carrying cells. Lack of evidence that the neu/erB2 oncogene was present or expressed in those normal ductal cells precludes deciding whether the mutated oncogene was necessary, sufficient, or merely irrelevant in these rare pre-carcinogenic events; moreover, the neu antigen was not visualized in all of the carcinomas in situ. We argue that these results are, in fact, consistent with the oncogene causing a change in cells in the epithelium that leads to a disruption of morphostats and that the disruption of morphostats is the proximal cause of cancer.

(6) A different type of evidence sometimes cited in support of the somatic mutation theory is the observation that the incidence of some types of cancer increases rapidly with age. Such a rapid increase could be explained by several genetic changes yet to be identified or by a few genetic changes interspersed with clonal expansion. The additional observation that various phenotypes must be acquired suggests multiple mutations with no clonal expansion or a mutator mutation (a mutation that generates more mutations) coupled with clonal expansion [41]. These observations could also be consistent with an initiation of cancer due to disruption of the morphostat gradient followed by mutations and/or clonal expansion. The increase in cancer incidence with age can also be explained by aging of normal tissue (which might affect the morphostat gradient). For instance, when tumors cells were injected into the livers of rats, the tumor cells regressed if the rats were young but progressed if the rats were old [42].

(7) Hereditary cancers (which account for about 5% of all clinical cancers) involve germ-line DNA mutations that could be considered as supporting the somatic mutation theory of carcinogenesis. An alternative explanation is that DNA mutations in these hereditary cancers are responsible for altered interactions among cells in a field where morphogens might play an important role during early development. The polyps in patients hemizygous for a defective APC (disrupting the wnt/wingless pathway) and the dysplasias preceding neoplasia both in retinoblastoma and in the lethal giant larva mutant in Drosophila that eventually result in neuroblastomas are tissue organization alterations in which the underlying cause may be altered morphogen/morphostat gradients [1].

### Problems with a hybrid theory

Under the somatic mutation theory, the proximal cause of cancer is one or more mutations in one or more cells [43,44]. The theory expounded here is that the proximal cause of cancer is a disruption of the morphostat gradient. Some confusion between the theories may arise from the fact that mutations have two different roles in the theory that the proximal cause of cancer is a disruption of the morphostat gradient. First, mutations are one possible cause of a disruption in morphostats (but there are other possible causes as well). Second, mutations are effects of the initiation of cancer. This is not equivalent to a hybrid theory.

## Conclusion

We hypothesize that the proximal step in the initiation of cancer is a disruption of a morphostat gradient. The argument that the oncogene experiments support *only *the somatic mutation theory does not account for the possibility that the most crucial component of the causal pathway could still require the disruption of morphostats. Although a mutation or multiple successive mutations in a single cell as the initial step cannot be ruled out, there are many experiments, such as the aforementioned foreign-body induced cancers, that are inconsistent with a mutation causing the initiation of cancer.

Our results challenge fundamental assumptions about early carcinogenesis and highlight the need to incorporate the study of stroma/epithelial interactions and morphogens/morphostats [2,3,10,45-48]. We hope that the results of this simulation will spur experimentalists to seek to identify relevant morphostats and test some fundamental assumptions about early carcinogenesis. In addition, the implications for the development of experimental systems are profound, suggesting that two-dimensional, and even three-dimensional, tissue-culture experiments that focus solely on epithelial cells could be missing a central mechanism of carcinogenesis.

## Competing interests

The authors declare that they have no competing interests.

## Authors' contributions

SGB wrote the software and the initial draft of the manuscript. BSK, AMS, CS, and AC provided substantive comments on the manuscript and the model. JP, building on earlier work including that from AMS and CS, formulated the morphostat hypothesis and provided substantive comments on the manuscript and the model. All authors have read and approved the final manuscript.

## Pre-publication history

The pre-publication history for this paper can be accessed here:

http://www.biomedcentral.com/1471-2407/9/89/prepub

## Supplementary Material

Additional file 1 Mathematical formulation of rules for cell renewal. The various rules for the movement and differentiation of cells are defined mathematically. Click here for file

Additional file 2 Software for computer simulation. A Mathematica package for the computer simulation is provided. Click here for file
