# Supplementary material for: Plausibility of stromal initiation of epithelial cancers without a mutation in the epithelium: a computer simulation of morphostats
Source: BMC Cancer. 2009 Mar 23;9:89. doi: 10.1186/1471-2407-9-89 (PMC2663766; doi:10.1186/1471-2407-9-89)
Supplement: Additional file 1 — Mathematical formulation of rules for cell renewal. The various rules for the movement and differentiation of cells are defined mathematically. [file 1471-2407-9-89-S1.doc]

**Appendix**

The cell types are coded as follows:

| Cell type | Bound-ary | Open space | Stem cell | Middle layer cell | Top layer cell | Stroma | Block | Aber-rant middle layer cell | Aberrant top layer cell | Lumen space |
| --- | --- | --- | --- | --- | --- | --- | --- | --- | --- | --- |
| Code | -1 | 0 | 1 | 2 | 3 | 4 | 5 | 6 | 7 | 8 |

Each cell corresponds to center cell type and adjacent cell types denoted by points on the compass:

| Notation for cell types | | |
| --- | --- | --- |
|  | n = north cell type |  |
| w = west cell type | c=center cell type | e = east cell type |
|  | s=south cell type |  |

Each cell corresponds to center morphostat level and adjacent morphostat levels, also denoted by points on the compass:

| Notation for morphostat levels | | |
| --- | --- | --- |
|  | n* = north level |  |
| w* = west level | c* = center level | e* = east level |
|  | s* = south level |  |

Let H denote the height of the tissue and W denote the width of the tissue, measure in number of cells. The horizontal position of the stem cell is set to the midpoint which equals the largest integer less than or equal to (W- 1)/2. Let K denote the number of cells of open space on top.

The initial gradient at the lower boundary (s=-1) is s*=M*=H-K. This yields a gradient of 0 at the top layer which occurs at vertical position H-K-1. Cells that are not stem or middle layer have a gradient set to -10 to indicate morphostat blockage.

A round consists of cell movement, lumen update, and morphostat update.

The cell movement rules are applied successively from the top to bottom rows. Within a row these rules can be applied either toward or away from the horizontal midpoint. On the final row above the stem cell, the rules are applied a number of times equal to half the number of cells in the row. This allows for all open spaces on that row to be replaced my new middle layer cells generated from stem cell.

The update of the morphostat gradient occurs from top to bottom and then can be specified to occur either from left to right or right to left. This update is repeated once per round to get closer to equilibrium.

Rule 1. *Sloughing of top-layer cell*

With probability p, a top layer cell (c=3) that is above a middle layer cell (s=2) and below lumen space (n=8) becomes open space (c_new_ =0).

| Original cell types | | |  | Revised cell types | | | |
| --- | --- | --- | --- | --- | --- | --- | --- |
|  | n=8 |  |  |  | n_new_=8 |  | |
| w | c=3 | e |  | w_new_=w | c_new_ =0 with probability p  c_new_ =3 with probability (1-p) | | e_new_=e |
|  | s=2 |  |  |  | s_new_=2 |  | |

Rule 2. *Upward movement of middle-layer cell*

When open space (c=0) or lumen space (c=8) occurs above a middle layer cell (s=2), the middle layer cell replaces the open space (c_new_=2), and the open space replaces the middle layer cell (s_new_=0), simulating upward movement of the middle layer cell.

| Original cell types | | |  | Revised cell types | | |
| --- | --- | --- | --- | --- | --- | --- |
|  | n=n |  |  |  | n_new_=n |  |
| w | c=0,8 | e |  | w_new_=w | c_new_ =2 | e_new_=e |
|  | s=2 |  |  |  | s_new_= 0 |  |

`

Rule 3. *Lateral movement of middle layer cell*

When there is an open space (c=0) not above a stem or middle layer cell (s1 and s2), lateral cell movement occurs according to one of two possible scenarios.

If the open space (c=0) is to the right of the stem cell (regardless of the row), a middle layer cell on the left is replaced by open space (w_new_=0), and the open space is replaced by the middle-layer cell (c_new_=2), simulating cell movement to the right.

| Original cell type to right of stem cell | | |  | Revised cell types | | |
| --- | --- | --- | --- | --- | --- | --- |
|  | n |  |  |  | n_new_=n |  |
| w=2 | c=0 | e=2 or e=-1 |  | w_new_=0 | c_new_ =2 | e_new_=e |
|  | s1 and s2 |  |  |  | s_new_=s |  |

If the open space (c=0) is to the left of the stem cell (regardless of the row, the middle layer cell on the right is replaced by open space (e_new_=0), and the open space is replaced by the middle-layer cell (c_new_=2), simulating cell movement to the left.

| Original cell type:  hoizontal position  midpoint | | |  | Revised cell types | | |
| --- | --- | --- | --- | --- | --- | --- |
|  | N |  |  |  |  |  |
| w=2 or w=-1 | c=0 | e=2 |  | w_new_=w | c_new_ =2 | e_new_=0 |
|  | s1, 2 |  |  |  | S1, 2 |  |

Rule 4. *Stem cell division*

When open space (c=0) occurs above a stem cell (s=1), a middle layer cell replaces the open space (c_new_=2) and the stem cell remains (s_new_=1), simulating the creation of a new middle layer cell from the stem cell.

| Original cell types | | |  | Revised cell types | | |
| --- | --- | --- | --- | --- | --- | --- |
|  | n=0 |  |  |  | n_new_=0 |  |
| W | c=0 | e |  | w_new_=w | c_new_ =2 | e_new_=e |
|  | s=1 |  |  |  | s_new_= 1 |  |

Rule 5. *Morphostat changes*

If the center cell is not a block, its revised morphostat level equals the maximum of the surrounding morphostat levels minus one ; otherwise its morphstat level is set to -10. The calculation includes an initial revision of surrounding morphostat levels depending on cell type. If the cell on the north, east, or west side is not a stem cell or middle-layer cell, its morphostat level is set to -10. If the cell on the south side is a stroma cell, its morphostat level is set to M*-1; if it is on the boundary its morphostat level is set to M*, and if it is another type of cell not a stem nor a middle-layer cell, its morphostat level is set to -10.

| Original morphostat level | | |  | Revised cell types | | |
| --- | --- | --- | --- | --- | --- | --- |
|  | n* |  |  |  | n_new_ |  |
| w* | c* | e* |  | w_new_ | c_new_ | e_new_ |
|  | s* |  |  |  | s_new_ |  |

| Revised morphostat levels | | |
| --- | --- | --- |
|  | n*_new_= n* if n_new_ = 0,1, 2,  n*_new_= 10, otherwise |  |
| w*_new_= w* if w= 0,1, 2,  w*_new_ = 10, otherwise | c*_new_ = max(n*, s*,e*,w*)1  if c  5,  c*_new_ = -10, if c=5 | e*_new_= e* if e=0, 1, 2,  e*_new_= 10, otherwise |
|  | s*_new_= s* if s=0, 1, 2,  s*_new_= M* if s= 1,  s*_new_= M*1 if f s= 4,  s*_new_ = 10, otherwise |  |

Rule 5 is applied from top to bottom and left to right (or vice versa) two times.

Rule 6. *Differentiation of middle-layer cell*

| Original cell type | New morphostat level | New cell type |
| --- | --- | --- |
| c=2 | c*_new_ =0 | c_new_ =3 |

Rule 7. *Formation of aberrant cell*

| Original cell type | New morphostat level | New cell type |
| --- | --- | --- |
| c=2 | c*_new_ <0 | c_new_ =6 |
| c=3 | c*_new_ <0 | c_new_ =7 |
